# Knee biomechanics changes under dual task during single-leg drop landing

**DOI:** 10.1186/s40634-019-0170-z

**Published:** 2019-02-07

**Authors:** Masaya Kajiwara, Akihiro Kanamori, Hideki Kadone, Yusuke Endo, Yasuto Kobayashi, Kojiro Hyodo, Tatsuya Takahashi, Norihito Arai, Yu Taniguchi, Tomokazu Yoshioka, Masashi Yamazaki

**Affiliations:** 1 Department of Orthopaedic Surgery, Faculty of Medicine, University of Tsukuba, Tsukuba, Japan; 2 Center for Innovative Medicine and Engineering, University of Tsukuba Hospital, Tsukuba, Japan; 3 Department of Physical Therapy, Ibaraki Prefectural University of Health Sciences, Ami-machi, Inashiki-gun Japan; 4 Department of Sport Management, Faculty of Business and Public Administration, Sakushin Gakuin University, Utsunomiya, Japan; 5 Musculoskeletal System, Department of Orthopaedic Surgery, Faculty of Medicine, University of Tsukuba, Tsukuba, Japan

**Keywords:** Single-leg drop landing, Dual task, Kinematics

## Background

Approximately 70% of anterior cruciate ligament (ACL) ruptures occur during single foot contact in sport (Boden et al. 2009; Olsen et al. 2004). Cohort studies that evaluated biomechanics during a vertical drop jump (VDJ) reported that this task could be used to screen for the risk of ACL rupture in athletes (Goetschius et al. 2012; Hewett et al. 2005; Padua et al. 2015). However, other studies found that exercise load from VDJ is too small to provide adequate assessment (Krosshaug et al. 2016; Reinschmidt et al. 1997). A variety of other assessment methods have been studied. Most three-dimensional motion analyses after ACL reconstruction have used low exercise load methods, such as gait or VDJ analyses, which are very easy for athletes who have returned to regular sports (Hall et al. 2012; Hooper et al. 2002; Ortiz et al. 2014).

Single-leg drop landing (SDL) is an assessment method with greater exercise load than VDJ, and studies comparing the two tasks found that knee load is significantly greater with SDL (Earl et al. 2007; Harty et al. 2011; Nagano et al. 2009; Pappas et al. 2007; Taylor et al. 2016), indicating that it is more suitable for examination of athletes. Studies found that knee kinematics do not differ significantly when healthy athletes perform SDL as a single task compared with VDJ (Ford et al. 2006; Wang 2011), suggesting that simply increasing exercise load is not sufficient to disturb the athlete balance and investigate knee biomechanics under conditions resembling competition; increasing the difficulty of the motion is also necessary.

In sports where ACL ruptures are common, such as basketball, handball, and soccer, athletes almost never decide their motion in advance, but are constantly moving in response to intense external disturbances, such as obstruction by opponents (Boden et al. 2000; Boden et al. 2009). Studies have used non-predictive tasks to take this into consideration, but most used low exercise load cutting motions or VDJ, and none performed SDL (Beaulieu et al. 2009; Besier et al. 2001; Herman and Barth 2016; Houck et al. 2006; Landry et al. 2007). These studies showed that subjects had a disturbed balance because non-predictive tasks extended the decision time and reduced preparation time for motion, changing kinematics. A dual task adds a neurocognitive load (via a cognitive task) to an exercise task, increasing the reaction time compared with a single task (Bekkering et al. 1994). Increasing reaction time also reduces preparation time for motion, and may be the reason for the disturbed balance of the athletes.

Therefore, the purpose of this study was to assess knee biomechanics among athletes during SDL under a dual task. The hypothesis of this study was that the maximum knee flexion angle, knee valgus angle, tibial internal rotation angle and anterior tibial translation and peak ground reaction force (GRF) during a dual task was larger than that of a single task during SDL.

## Methods

### Subjects

The subjects were 20 athletes (10 male and 10 female). The mean age of subjects was 20.0 ± 1.1 years, height was 167.3 ± 10.1 cm, weight was 64.0 ± 8.8 kg, and body mass index was 22.8 ± 1.8 kg/m^2^. All subjects were competitive-level players (17 soccer, 3 handball) from university. Athletes with a history of lower limb surgery or lower limb injury within the last 6 months, or skin disorders preventing the attachment of markers were excluded.

### Exercise task

**Fig. 1:**
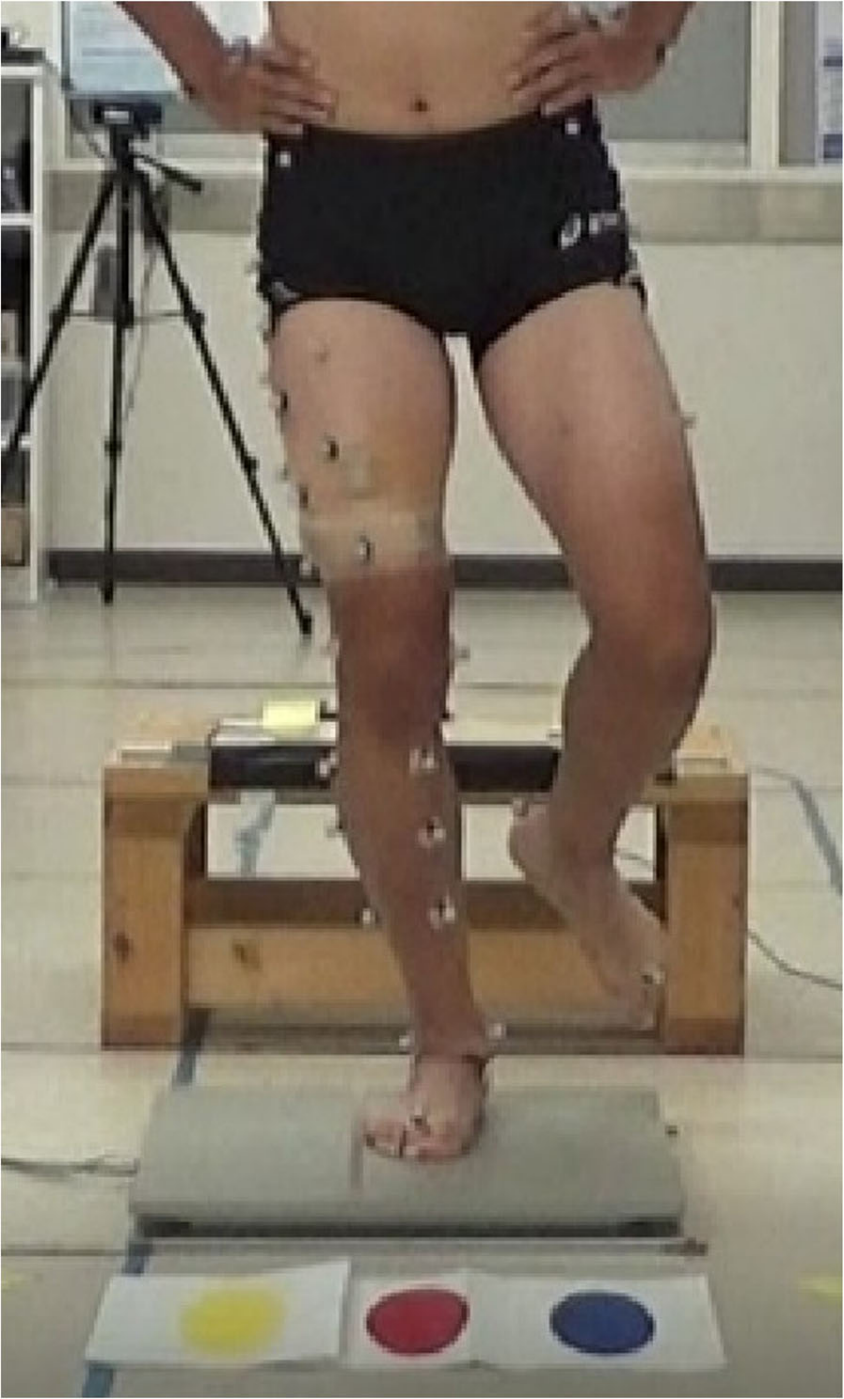
The subject jumps from a 30-cm platform onto a footplate. The landing site is one of three colors displayed on a monitor in front of the subject

With their hands on iliac crests, the subjects performed SDL barefoot from a 30-cm platform onto a force-plate (Accugait, AMTI Inc., Watertown, USA). Prior to measurements, the subjects warmed up and practiced the technique until accustomed to it. After practice, all subjects performed SDL under single-task conditions, and then performed SDL under dual task conditions. A footswitch (Scythe Co., Ltd., Matsudo, Japan) on the platform and a monitor (15.6 in., Lenovo, Hong Kong, China) were connected by a USB cable, with the monitor placed 3 m in front of the platform at a height of 30 cm. The monitor displayed the instruction immediately after activated by the heel left the footswitch. The task was successful if the subject landed on one of three spots shown on the monitor and remained stationary for 2 s (Fig. 1). Subjects performed at their own pace and when not fatigued. Failing to remain stationary for 2 s or removing hands from iliac crests were considered failures. The task was repeated until successfully performed three times.

### Cognitive task

The Stroop task is a frequently used cognitive task. Briefly, this task used a computer monitor displaying the words “blue”, “red”, or “yellow”; each word was displayed in a font color different to the meaning of the word. Subjects were told to respond to the color of the text, not its meaning. Instructions on where to land on the force-plate were given using these three colors and words. Adding the cognitive load of the Stroop task to another task was shown to increase reaction time relative to a single task performance (Bekkering et al. 1994). Single task conditions consisted of the subject performing an SDL without the Stroop test, while dual task conditions consisted of the subject performing an SDL with the Stroop test.

### Data measurement

Using a three-dimensional motion analyzer (Vicon MX, Vicon Motion System Inc., Oxford, UK), the coordinates of infrared reflective markers attached to subjects’ bodies were recorded between 40 ms before and after foot contact. A previous study showed that ACL ruptures occur within 40 ms (Koga et al. 2010), so this timeframe was used for kinematic analyses in this study. The reference position for these measurements was obtained during the static standing trial. The moment the GRF appeared was considered as the landing. Maximal knee angular displacements (flexion–extension, abduction–adduction, internal–external rotations), anterior tibial translation, and peak GRF were compared between the single and dual task. Sixteen infrared cameras (MX-T20, Vicon Motion System Inc., Oxford, UK) surrounded the subject, and the sampling frequency was set to 100 Hz. Twenty-three markers (14 mm) were attached following a point cluster technique (Andriacchi et al. 1998). A force-plate was synchronized with this system, and GRF during each movement was measured at a 1000-Hz sampling frequency. Wireless surface electrodes (Trigno Lab, Delsys, Inc., Boston, USA) were also synchronized with the three-dimensional motion analyzer to measure electromyographic activity of six muscles (vastus medialis, rectus femoris, vastus lateralis, semitendinosus, biceps femoris, and gluteus medialis). Electromyographic activity was evaluated as the integral during the 40 ms before and after initial foot contact. Sites of surface electrode attachment followed a previous study (Rainoldi et al. 2004). The markers and surface electrodes were only attached to the leg being measured.

### Data analysis

For each trial, three knee angular displacements (flexion–extension, abduction–adduction, internal–external rotations) and anterior tibial translation were calculated according to the joint coordinate system using the point cluster technique (Andriacchi et al. 1998). This technique can compute shank orientation within error of 0.37 degrees using skin surface marker positions (Alexander and Andriacchi 2001). Each maximum range of motion was analyzed. All dependent variables were calculated for each trial, and then averaged across the three trials.

### Statistical processing

The SDL was performed in a single and dual task. A t-test was used to compare the two tasks. All statistical comparison was performed with the level of significance set at *p* < 0.05.

### Ethical considerations

This study design was approved by the University of Tsukuba’s institutional review board (H28–188). All subjects provided written informed consent before participation.

## Results

### Kinematics data

**Fig. 2:**
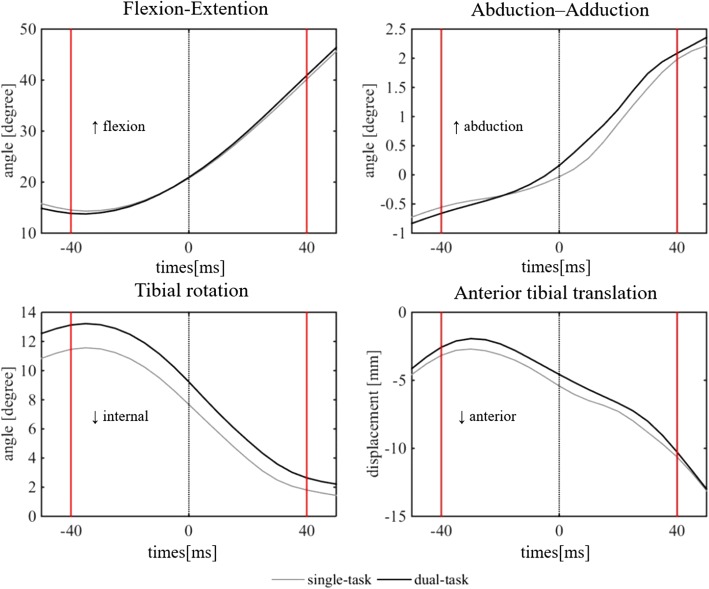
Time series curves for the mean three knee angular displacements (flexion–extension, valgus–varus, internal–external rotation) and the anterior tibial translation during the 40 ms before and after initial foot contact for the two tasks (single and dual task)

The peak GRF was evaluated as the integral between 0 ms and 40 ms after initial foot contact. It was 3.7 ± 0.7 Nm/kg and 4.1 ± 0.7 Nm/kg for the single and dual task, respectively. Peak GRF was 0.4 Nm/kg (11.8%) greater in the dual task (*p* < 0.05). Peak GRF varied between subjects, but the mean timing was 42 ms after initial foot contact. Figure 2 shows the time series curves for the mean three knee angular displacements (flexion–extension, valgus–varus, internal–external rotation) and the anterior tibial translation in the 40 ms before and after initial foot contact under single and dual tasks. Table 1 shows the means and SDs of three angular displacements and the anterior tibial translation comparison between the two tasks. The maximum tibial internal rotation angle was significantly larger (15.2%, *p* < 0.05) in the dual compared with single task (13.8 ± 4.8 vs 11.9 ± 4.7 and degrees, respectively.. The maximum knee flexion angle, knee valgus angle, and anterior tibial translation were not significantly different between the two tasks.

**Table 1:**
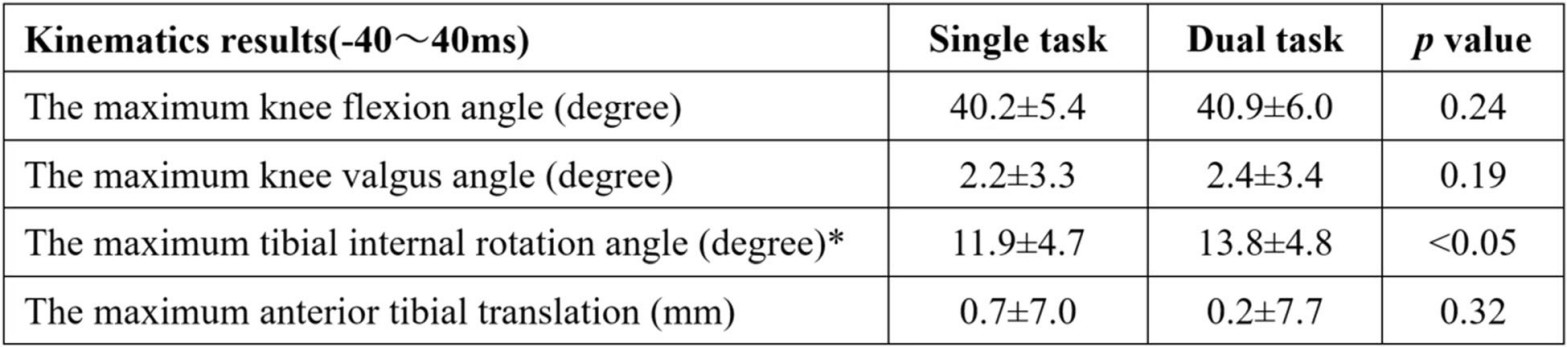
Kinematics results comparing the single and dual task. Values are given as mean ± SD

* indicate a significant difference between the single and dual task (*p* < 0.05).

### Electromyographic data

Table 2 shows the results for the integral electromyographic activity for the six tested muscles. In this study, rectus femoris activity tended to be higher relative to hamstring activity, but the difference was not significant between single and dual tasks. These results demonstrate that the other muscles were not significantly different when compared between the two tasks.

**Table 2:**
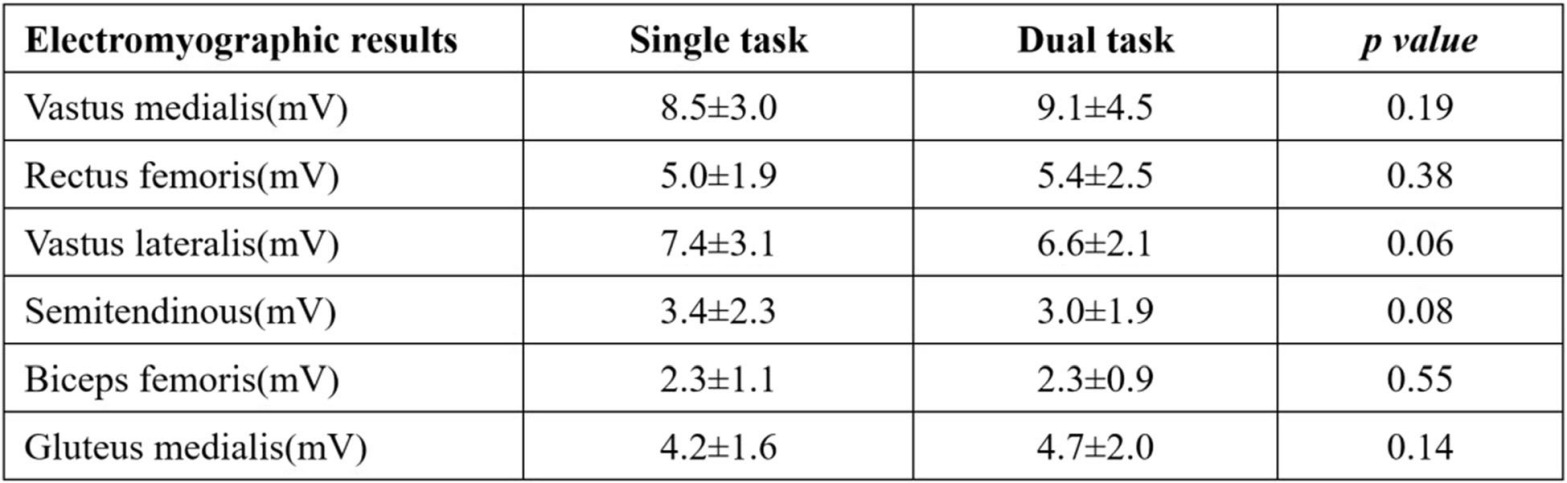
Muscle activity results comparing the single and dual tasks. Values are given as mean ± SD

## Discussion

The most important findings of this study were that the maximum tibial internal rotation angle and peak GRF increased under dual task conditions. In this study, we used the point cluster technique to assess knee biomechanics of athletes while performing SDL as a single or dual task.er dual task conditions. Our hypothesis had five candidate factors that may increase ACL rupture risk, and we show two of five factors increased under the dual task.

In vivo studies have shown that ACL strain increases the motion of knee flexion, tibial internal rotation, knee valgus, and anterior tibial translation (Asano et al. 2001; Kiapour et al. 2016). These studies demonstrated maximum ACL strain occurs during multiplanar loads of these four motions. In the current study, the maximum angle at landing was increased for tibial internal rotation only. Using the point cluster technique, Nagano et al. previously demonstrated that knee valgus angle and tibial internal rotation angle increased during SDL in a single task (Nagano et al. 2009); however, subjects were not athletes. We found that the maximum tibial internal rotation angle increased but knee valgus angle did not increase. A previous study showed that ACL strain increased because of changes in knee kinematics (Koga et al. 2010). The greater the changes in knee kinematics, the more the ACL strain increases, which can lead to ACL rupture.

High-risk sports for ACL rupture include basketball, handball, and soccer (Boden et al. 2000). Video analyses of these sports show that injuries occur when players are focused on the goal or an opponent instead of their own bodies (Boden et al. 2009; Hewett et al. 2009). Many studies of SDL as a single task indicated changes in knee kinematics, but some studies showed that knee valgus and tibial internal rotation angle did not differ significantly in healthy athletes performing SDL or VDJ as single tasks (Ford et al. 2006; Wang 2011). The results of these studies may indicate that even if exercise load is increased, disturbing the balance of athletes is unlikely in conditions where they can focus on their own motion.

Research has shown that when healthy athletes are given a non-predicted random task, knee valgus and tibial internal rotation angles increase, thus increasing the risk of ACL rupture (Besier et al. 2001; Houck et al. 2006; Landry et al. 2007). However, these investigations were not conducted using the point cluster technique; limiting their evaluation of knee biomechanics, and in particular the assessment of internal and external rotation. In addition, past investigations were not conducted using SDL under dual task conditions.

The results of previous studies suggested that the increased injury risk during non-predicted tasks may be due to the short time available to prepare for motion (Herman and Barth 2016). Non-predicted tasks prolong decision time, which concomitantly shortens the time to prepare for motion and compromises balance during the task. In contrast, when performing a single task such as the SDL, athletes have sufficient time to prepare for the movement because there is no decision time and their balance is not compromised (Ford et al. 2006; Wang 2011). Thus, our current study assessed changes to knee kinematic during SDL under single task compared with dual task conditions. The kinematic changes increasing the maximum internal tibial rotation angle are caused by a similar mechanism, as a longer decision time is necessary for non-predicted tasks. Many studies have indicated that the Stroop task prolongs reaction time compared with a simple non-predicted task (De Marchis et al. 2013; Washburn et al. 2016). In this study, we assessed knee biomechanics in athletes performing simultaneous exercise and cognitive tasks, creating a more difficult task than the single non-predicted task used in other studies.

Increased force generation by the rectus femoris leads to anterior tibial translation (Sheehan et al. 2012; Beaulieu et al. 2012) and tibial internal rotation (DeMorat et al. 2004). Therefore, rectus femoris activity increases the load on the ACL. We predicted that rectus femoris activity under dual task conditions would be greater than under single task conditions. Although rectus femoris activity was higher in the dual compared with single task, the difference was not significant. It is possible that there was no unnecessary activation of muscles, as athletes focused on the cognitive task and not their own bodies during dual task conditions.

There are several limitations in this study. First, the study included a small number of subjects. The reliability of data would increase if more subjects were included. Second, knee biomechanics were only analyzed using a point cluster technique. Previous studies have found that ankle and hip biomechanics are also associated with risk for ACL rupture (McLean et al. 2004; Zazulak et al. 2005). Therefore, the biomechanics of other joints during dual task need to be evaluated in the future studies. Third, there may be a limited learning effect in the SDL. All athletes practiced the SDL technique prior to measurements, and then performed single tasks first, followed by dual task conditions. As a result, there may be some learning effect in the subsequent dual task conditions, as the athletes may be more accustomed to the movements.

## Conclusion

The results of this study demonstrate that dual task using SDL instructions increased tibial internal rotation angle and peak GRF. This suggests that motions that are combined with a cognitive task are associated with higher tibial internal rotation angle and peak GRF.

## Abbreviations

ACL: Anterior cruciate ligament
GRF: Ground reaction force
SDL: Single-leg drop landing
VDJ: Vertical drop jump
